# A Cancer Risk Assessment of Inner-City Teenagers Living in New York City and Los Angeles

**DOI:** 10.1289/ehp.8507

**Published:** 2006-06-15

**Authors:** Sonja N. Sax, Deborah H. Bennett, Steven N. Chillrud, James Ross, Patrick L. Kinney, John D. Spengler

**Affiliations:** 1 Gradient Corporation, Cambridge, Massachusetts, USA; 2 University of California–Davis, Davis, California, USA; 3 Lamont Doherty Earth Observatory and; 4 Mailman School of Public Health, Columbia University, New York, New York, USA; 5 Harvard School of Public Health, Boston, Massachusetts, USA

**Keywords:** aldehydes, cancer risk assessment, metals, personal exposures, VOCs

## Abstract

**Background:**

The Toxics Exposure Assessment Columbia–Harvard (TEACH) project assessed exposures and cancer risks from urban air pollutants in a population of high school teenagers in New York City (NYC) and Los Angeles (LA). Forty-six high school students participated in NYC and 41 in LA, most in two seasons in 1999 and 2000, respectively.

**Methods:**

Personal, indoor home, and outdoor home 48-hr samples of volatile organic compounds (VOCs), aldehydes, particulate matter with aerodynamic diameter ≤ 2.5 μm, and particle-bound elements were collected. Individual cancer risks for 13 VOCs and 6 particle-bound elements were calculated from personal concentrations and published cancer unit risks.

**Results:**

The median cumulative risk from personal VOC exposures for this sample of NYC high school students was 666 per million and was greater than the risks from ambient exposures by a factor of about 5. In the LA sample, median cancer risks from VOC personal exposures were 486 per million, about a factor of 4 greater than ambient exposure risks. The VOCs with the highest cancer risk included 1,4-dichlorobenzene, formaldehyde, chloroform, acetaldehyde, and benzene. Of these, benzene had the greatest contributions from outdoor sources. All others had high contributions from indoor sources. The cumulative risks from personal exposures to the elements were an order of magnitude lower than cancer risks from VOC exposures.

**Conclusions:**

Most VOCs had median upper-bound lifetime cancer risks that exceeded the U.S. Environmental Protection Agency (EPA) benchmark of 1 × 10^−6^ and were generally greater than U.S. EPA modeled estimates, more so for compounds with predominant indoor sources. Chromium, nickel, and arsenic had median personal cancer risks above the U.S. EPA benchmark with exposures largely from outdoors and other microenvironments. The U.S. EPA–modeled concentrations tended to overestimate personal cancer risks for beryllium and chromium but underestimate risks for nickel and arsenic.

The health risks associated with exposures to common urban air pollutants have focused primarily on the six U.S. Environmental Protection Agency (EPA) criteria air pollutants. Much less is known about exposures and adverse health impacts of the mix of > 100 hazardous air pollutants (HAPs) identified by the U.S. EPA in the 1990 Clean Air Act Amendments as posing health risks to the general population. One important chronic health impact associated with exposures to HAPs is cancer. Of the 188 HAPs, 91 compounds are known, probable, or suspected carcinogens with available inhalation unit risks, including many volatile organic compounds (VOCs) (Woodruff et al. 2000).

Most individuals are exposed to HAPs while indoors (residence, workplace, school, and vehicles), where people spend most of their time. Indoor concentrations of HAPs, however, can originate from outdoor as well as indoor sources. Regulatory efforts by the U.S. EPA have focused primarily on ambient concentrations (emissions, measurement, and modeling). For example, in 1995 the U.S. EPA undertook the Cumulative Exposure Project (CEP), modeling concentrations of 148 HAPs using emissions data in conjunction with an air dispersion model, the Assessment System for Population Exposure Nationwide (Woodruff et al. 1998). The results from the CEP modeling efforts showed that in both New York City (NYC) and Los Angeles (LA), cancer risks of most VOCs exceeded the U.S. EPA benchmark (1 × 10^−6^ risk for a lifetime) by at least a factor of 2 but as much as a factor of 100 for some compounds (Caldwell et al. 1998). More recently, the U.S. EPA conducted the National Air Toxics Assessment (NATA) using emissions data from 1996 for 33 priority HAPs, most of them VOCs (U.S. EPA 2001).

These efforts by the U.S. EPA help characterize HAP concentrations, model trends over time, and help prioritize research and regulatory actions. However, ambient concentrations fail to account for additional exposures from indoor sources. The U.S. EPA has recognized these limitations and is currently developing an exposure model that includes exposures indoors and in other microenvironments (U.S. EPA 2001). However, the dearth of studies on indoor and personal concentrations of HAPs has hindered the development of accurate exposure models. Studies that have addressed personal exposures show a general trend of personal concentrations exceeding indoor concentrations, which in turn exceed ambient levels. In addition, interpersonal variation of personal HAP exposures depends on activity patterns and type of indoor environments encountered (Adgate et al. 2004; Akland 1993; Calderon et al. 2003; Caussy et al. 2003; Chillrud et al. 2004; Clayton et al. 1999; Wallace 1987; Wallace et al. 1988, 1989; Weisel 2002).

The Toxics Exposure Assessment Columbia–Harvard (TEACH) project collected data on personal, indoor, and outdoor concentrations of various HAPs, including a suite of VOCs, aldehydes, and particle-bound elements, with the goal of determining levels of exposure and potential cancer risks among a sample of urban teenagers (Kinney et al. 2002). The study population consisted of inner-city high school students living in NYC and LA. Two-day samples were collected in NYC and LA in two seasons. In this article, we present estimated cancer risks associated with personal exposures to VOCs and particle-bound elements, apportioned to indoor home and outdoor home microenvironments. In addition, we present the contribution to the total personal cancer risk from each of the compounds. Finally, personal exposure estimates are compared with U.S. EPA–modeled ambient estimates.

## Materials and Methods

We recruited nonsmoking teenagers (13–19 years of age), from nonsmoking homes, from the A. Philip Randolph High School in west Harlem in NYC and the Jefferson High School in south central LA. Recruitment details are described elsewhere (Kinney et al. 2002). The protocol was approved by the Columbia Health Sciences Institutional Review Board and the Harvard Human Subject Committee. Also, all of the participants and their parents or guardians signed consent forms before involvement in the study.

A personal sample, home indoor sample, and home outdoor sample were collected for each participant. Sampling was conducted in two seasons, winter (February–April 1999) and summer (June–August 1999) in NYC and winter (February–March 2000) and fall (September–October 2000) in LA. The seasons were chosen to try to maximize the potential differences in pollutant concentrations, with higher concentrations typical in winter and lower concentrations in summer/fall for most VOCs and particle-bound elements in this analysis (Sax et al. 2004).

In NYC, 46 individuals were sampled, 38 in winter and 41 in summer, with 33 subjects monitored in both seasons. Most of the homes were located in upper Manhattan and the Bronx (> 80%) and the rest in the Brooklyn and Queens boroughs of NYC. In LA, we had 40 winter participants and 35 fall participants, of whom 34 were sampled in both seasons. All homes located in south central LA were within 5 km (3.1 miles) of the school.

The personal sampler was run by a BGI pump (BGI Inc., Waltham, MA) with the flow split three ways to collect particulate matter with aerodynamic diameter ≤ 2.5 μm (PM_2.5_) on a Teflon filter at 4 L/min, VOCs with a thermal desorption tube at 1.8 standard cc^3^/min, and aldehydes using a 2,4-dinitro-phenylhydrazine (DNPH)–coated C_18_ sampler at approximately 100 standard cc^3^/min. This personal sampler was housed in a customized backpack that the students carried over their shoulders. The indoor monitor was typically located in the living room, and the outdoor sampler was set up to monitor through a window. Two sampling boxes containing three 7 L/min pumps (Medo, Inc., Hanover Park, IL) were used to collect samples inside and outside of each subject’s home, as described previously (Sax et al. 2004). Each week of the campaign three to five participants were sampled. A sampling session consisted of a 48-hr period only on weekdays, typically Tuesday through Thursday.

Target VOCs were collected on multisorbent “Air Toxics” tubes (PerkinElmer, Norwalk, CT). The sampling and analytical methods are described in U.S. EPA’s compendium method TO-17 (U.S. EPA 1999a; Woolfenden and McClenny 1997). Analysis of VOC tubes was carried out using a PerkinElmer model 400 automatic thermal desorber connected to a Hewlett Packard model 5890II gas chromatograph and model 5971 mass selective detector (Hewlett Packard Co., Palo Alto, CA). Aldehydes were sampled using the methodology described in the U.S. EPA’s compendium method TO-11A (U.S. EPA 1999b), with air pumped through a C_18_ cartridge coated with acidified DNPH. The coated samplers were obtained from AtmAA, Inc. (Calabasas, CA). The DNPH derivatives (hydrazones) were eluted with acetonitrile and then analyzed using high-performance liquid chromatography with a Hewlett-Packard 1100 and ultraviolet detection at 360 nm.

Field blanks were used to determine background contamination and to calculate limits of detection (LODs). LODs were generally ≤ 1 μg/m3 except for methylene chloride, benzene, 1,4-dichlorobenzene, and toluene for select cities and seasons. We calculated the mean relative difference (MRD) as a measure of precision by taking the absolute difference of a pair of duplicates divided by the mean of the pair. For most compounds, the MRD was < 25%; 1,3-butadiene had the highest MRD (41%). Details can be found elsewhere (Sax et al. 2004). VOC and aldehyde breakthroughs were tested using backup tubes, and concentrations were indistinguishable from blanks. Samples lost because of equipment or analytical problems were excluded from data analysis. All concentrations were blank corrected, and negative values were set to zero.

PM_2.5_ was collected on Teflon filters housed in plastic cassettes attached downstream from a BGI cyclone with a 2.5-μm cut point when operated at 4 L/min ± 10%. Filters were prepared for determination of 28 elements by magnetic-sector high-resolution inductively coupled-plasma mass spectrometry (HR-ICP-MS). Diluted digests were analyzed by HR-ICP-MS for all isotopes of interest at the appropriate resolving power to avoid isobaric interferences. Winter NYC samples were run on a Finnegan Element [Finnigan-Mat, Bremen, Germany (now Thermo Electron Co., Waltham, MA)]; all other digests were run on an Axiom [VG-Elemental, Winsford, UK (now Thermo Electron)]. Detailed analytical methods can be found elsewhere (Chillrud et al. 2004). Quantification was done by external and internal standardization. Reproducibility of field blank samples (3× SD) was used to derive sample detection limits. No chromium, beryllium, and arsenic data are reported for NYC winter because of high procedural blanks. Aliquots of standard reference material (SRM) 1648 (urban particulate matter) from the National Institute of Standards and Technology (Gaithersburg, MD) were digested and analyzed in the same manner as the samples several times during the course of the sample analyses. Recoveries for most analytes were within 10% of reported values for the SRM and within 20% for all reported analytes. Precision estimates, based on the median percent difference of pairs of duplicate samples, were 20% or better for most analytes, with chromium the exception at about 30%.

We averaged personal, indoor, and outdoor concentrations across seasons if the subject had measurements for both seasons; if not, then data from a single season were used. For example, in NYC for 1,4-dichlorobenzene we had a total of 66 measurements, of which 50 came from paired winter–summer samples (representing 25 participants) and the remaining 16 were unmatched from 16 individuals, for a total of 41 individuals.

We used inhalation unit risk factors representing the probability that an individual will develop cancer as a result of exposure to 1 μg/m^3^ of the compound over a lifetime (70 years). They are typically nonthreshold linear, high-dose to low-dose extrapolations from animal or occupational studies. The unit risks either are calculated by using maximum-likelihood estimates from a dose–response relationship or represent the 95% upper-bound estimate. Unit risk values were taken from the Integrated Risk Information System (U.S. EPA 2005b) when available and alternatively from the California Environmental Protection Agency (CalEPA 2002). We determined the cumulative risks by adding across the target VOCs.

The contribution to total personal cancer risk from indoor and outdoor sources was calculated by using time-weighted concentrations of indoor and outdoor home concentrations, using the following model:


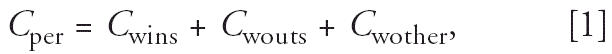


where


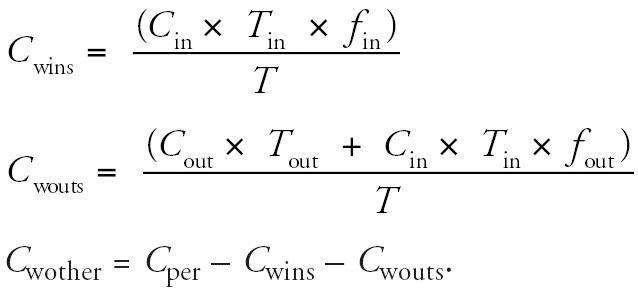


*C*_per_ is the measured personal concentration (micrograms per cubic meter); *C*_wins_ is the time-weighted indoor concentration from indoor sources (micrograms per cubic meter); *C*_wouts_ is the time-weighted concentration from outdoor sources, including both exposure occurring outdoors and exposure occurring indoors at home attributable to outdoor sources (micrograms per cubic meter); *C*_wother_ is the time-weighted concentration from other microenvironments (micrograms per cubic meter). *C*_in_ is the concentration measured in each home (micrograms per cubic meter); *C*_out_ is the concentration measured outside the home (micrograms per cubic meter); *T* is the total sampling time (min); *T*_in_ is the time spent indoors (minutes); *T*_out_ is the time spent outdoors (anywhere) (minutes); *f*_in_ is the fraction of indoor concentration from indoor sources; and *f*_out_ is the fraction of indoor concentration from outdoor sources.

The time spent indoors at home and outdoors was taken from time–activity questionnaires filled out by each participant. We determined indoor concentrations from indoor and outdoor sources by a mass balance model using indoor and outdoor concentrations, home volume, and air exchange rates. For VOCs, penetration rate was set equal to 1 and deposition rate to 0 (Sax et al. 2004). For the particle-bound elements, we determined the penetration rate using the indoor–outdoor sulfur ratio. The time-weighted contribution from outdoor sources includes both outdoor exposures and indoor exposures from outdoor origin.

As indicated by *C*_wother_ in Equation 1, the concentration in other microenvironments is not measured but rather calculated as the difference between personal exposure and exposure attributable to both indoor home and outdoor microenvironments. This method possibly underestimates total contribution from outdoor sources because concentrations in “other” microenvironments are influenced by indoor and outdoor sources, and we do not apportion this exposure.

## Results

### Subject demographics

In NYC, 46 self-reported nonsmoking students from nonsmoking homes were enrolled. Subjects’ age ranged from 14 to 19 years, with 31 (67%) female and 15 (33%) male participants. The racial distribution was 43% African American, 50% Hispanic, and the remaining 7% either Asian or not reported. These and other sociodemographic characteristics were similar to a larger group of 611 students surveyed at the school (Sax 2003), except that the percentage of females was higher in our sample (67%) than in the surveyed population (58%). The NYC high school is a science and math magnet school drawing gifted students from across the city. In LA, of the 41 students that participated, 13 (33%) were male, 27 female (67.5%), and one had missing data. Most were Hispanic (93%) with one African American and one American Indian, reflecting the neighborhood demographics. Ages ranged from 13 to 17 years. The demographics of enrolled students were similar to those of the 733 students surveyed in the same school (Sax 2003), except we enrolled more female students.

### Exposures and cancer risks

Descriptive statistics (median, mean, and maximum values) of the pooled personal, indoor, and outdoor concentrations are presented in Table 1, which shows the total number of participants and in parentheses the number of participants with winter and summer/fall paired data. The percentage of samples above the LOD was greatest for personal samples, with most compounds detected in > 90% of samples. The exceptions were 1,3-butadiene and nickel, which had > 60% detects in NYC, and in LA, 1,3-butadiene and trichloroethylene (TCE), which had > 80% detects. Median personal concentrations of VOCs ranged from < 0.5 μg/m^3^ for TCE in both NYC and LA, and for 1,3-butadiene and chloroform in LA, to between 10 and 20 μg/m^3^ for acetaldehyde, formaldehyde, and methyl-*tert* butyl ether (MTBE) in both cities. A much larger range of concentrations was found for the elements, spanning almost 5 orders of magnitude, from < 0.003 ng/m^3^ for beryllium to 18 ng/m^3^ for nickel. In NYC, several VOCs had maximum personal concentrations > 100 μg/m^3^, including tetrachloroethylene, methylene chloride, MTBE, and 1,4-dichlorobenzene, whereas in LA only 1,4-dichlorobenzene ever exceeded 100 μg/m^3^. As expected, for most VOCs, median personal concentrations were equal to or greater than median indoor concentrations and at least twice as high as median outdoor concentrations. The exceptions were carbon tetrachloride and MTBE in NYC and LA, and ethylbenzene and benzene in LA. In NYC, similar concentrations across indoor, outdoor, and personal environments were seen for most of the PM_2.5_ particle-bound elements, except for chromium. Mean personal concentrations of chromium were twice as high as indoor home concentrations and about three times higher than outdoor home concentrations. In LA, mean personal concentrations of beryllium were two times higher than indoor and outdoor levels, and nickel concentrations were about four times higher compared with indoor and outdoor concentrations.

**Figure 1:**
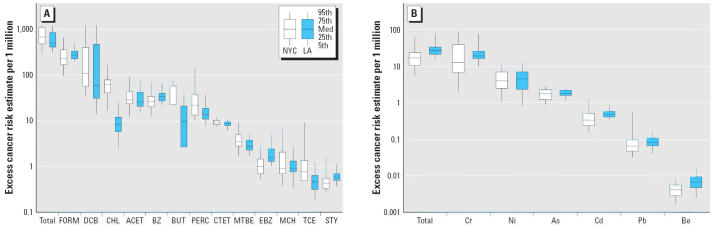
Distribution of excess upper-bound cancer risks based on personal concentrations of TEACH teenagers in NYC and LA for VOCs (*A*) and elements (*B*). Cancer risks are on a log scale and are expressed as excess cancers per million population. Med, median. 5th, 25th, 75th, and 95th are percentiles. Abbreviations for VOCs: ACET, acetaldehyde; BUT, 1,3-butadiene; BZ, benzene; CHL, chloroform; CTET, carbon tetrachloride; DCB, 1,4-dichlorobenzene; EBZ, ethylbenzene; FORM, formaldehyde; MCH, methylene chloride; PERC, tetrachloroethylene; STY, styrene; Total, cumulative risks from all VOCs. Abbreviations for elements: As, arsenic; Be, beryllium; Cd, cadmium; Cr, chromium; Ni, nickel; Pb, lead; Total, cumulative risks from all elements.

Cancer risk estimates calculated based on measured personal concentrations are presented in Table 2. Risks are expressed as excess cancers per 1 million population based on exposures over a 70-year lifetime. Mean VOC cumulative risks were 957 per million population in this sample of NYC high school students (median, 666) compared with 806 in this sample of LA high school students (median, 486). The cumulative VOC risks for individual participants spanned from about 100 to > 4,100 excess cancers per million population in both cities’ teenage samples. In contrast, the mean cumulative risks for the elements were 34 per million population for both cities’ teenagers (median, 23 and 26 in NYC and LA, respectively), with a range of 3–173 per million over different subjects. The VOCs with the highest risks in both cities were formaldehyde and 1,4-dichlorobenzene (Table 2). In comparing the two samples of teenagers, NYC teenagers had significantly higher risks from chloroform (median, 61 vs. 8) and tetrachloroethylene (median, 22 vs. 13) than did those in the LA sample. Also, 1,3-butadiene risks were greater in NYC teenagers, although fewer samples were above the LOD for 1,3-butadiene in the NYC sample. Other VOCs had similar risks between the two cities. Figure 1 summarizes the risks across compounds for the two cities. The box plots indicate the 5th, 25th, 50th, 75th, and 95th percentiles of cancer risks for individual compounds and total cumulative risk based on personal concentrations.

**Figure 2:**
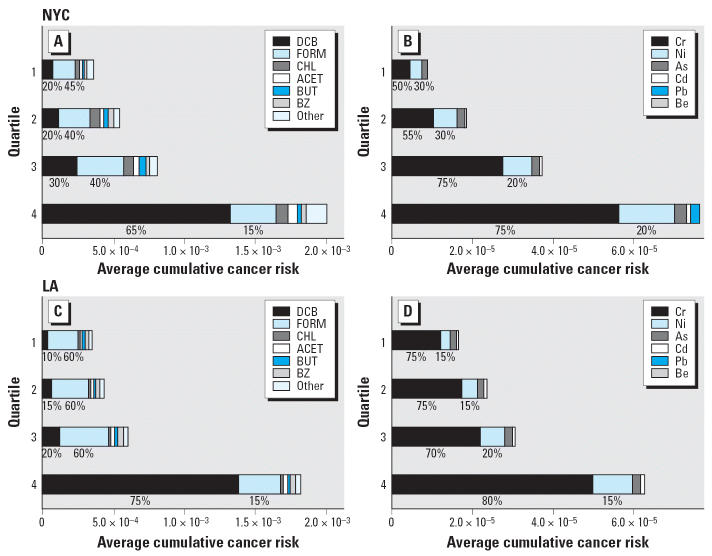
Distribution of upper-bound excess cancer risks averaged over quartiles of individuals for VOCs (*A* and *C*) and elements (*B* and *D*) in NYC and LA, respectively. Each quartile represents the average contribution to risk from each pollutant for approximately 10 teenagers. Risks are rank ordered from highest cumulative risk (quartile 4) to lowest cumulative risk (quartile 1). Abbreviations for VOCs: ACET, acetaldehyde; BUT, 1,3-butadiene; BZ, benzene; CHL, chloroform; DCB, 1,4-dichlorobenzene; FORM, formaldehyde; Other, carbon tetrachloride, tetrachloroethylene, ethylbenzene, methylene chloride, TCE, and styrene. Abbreviations for elements: As, arsenic; Be, beryllium; Cd, cadmium; Cr, chromium; Ni, nickel; Pb, lead.

Although formaldehyde and 1,4-dichlorobenzene were the largest contributors to risk overall, not all teenagers had the same percent contribution to total risk from these compounds. Figure 2 shows quartiles of teenagers rank ordered from lowest (quartile 1) to highest cumulative risk (quartile 4), with the mean percent contribution from each compound averaged within the quartile. For the teenagers in the high-risk quartile (quartile 4), 1,4-dichlorobenzene accounted for 65% of the total risk in NYC and 75% in LA, whereas in the lowest quartile, formaldehyde accounts for 45% of the risk in NYC and 60% in LA, and 1,4-dichlorobenzene was only 10–30% of the risk. Chromium is the element contributing most to the overall risk from particle-bound elements in both NYC and LA. In NYC, chromium accounts for a much larger fraction (75%) of the risk in the upper 50th percentile (quartiles 3 and 4) of the population, but in LA the contributions to personal risk were relatively constant across quartiles (70–80%).

### Microenvironmental contributions to risk

To examine the contributions to personal risk from home indoor sources, outdoor sources, and other microenvironments, concentrations were time-weighted using individual time–activity data. Further, indoor concentrations were apportioned to determine the fraction from outdoor and indoor sources. Exposures not accounted for by indoor home or outdoor sources were classified as “other” and could include exposures in the school or other indoor environments (both the indoor and outdoor contributions to these environments) and commuting. The time–activity results are summarized in Table 3. An average of 18 hr was spent at home each day. Teenagers spent the second largest portion of their day at school. In NYC, all the teenagers were in school in the winter and were off in the summer, whereas in LA some teenagers attended school during both the fall and winter sampling periods. In LA, some students were out of school during one or both of the sampling seasons due to a year-round multitrack school system. Note that all sampling days were weekdays.

**Figure 3:**
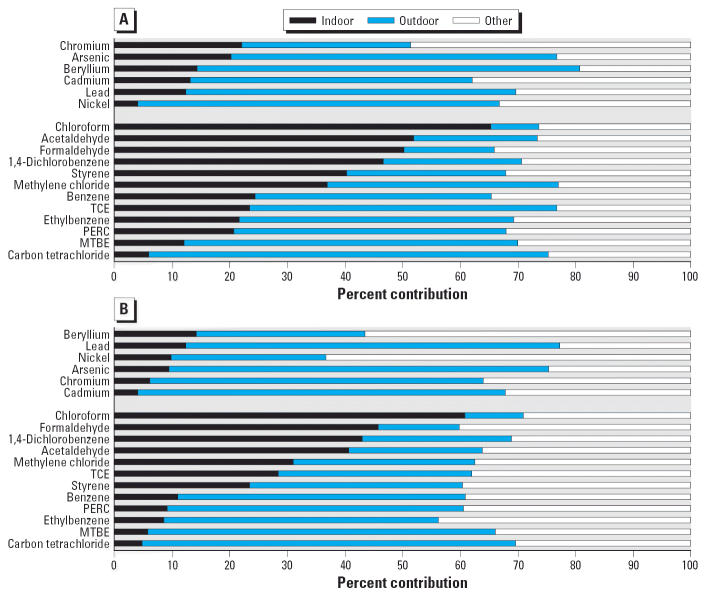
Mean percent contributions to personal cancer risks from home indoor, outdoor, and other microenvironments in (*A*) NYC and (*B*) LA. PERC, tetrachloroethylene.

Figure 3 summarizes the percent contributions from the indoor, outdoor, and other microenvironment sources for mean personal risks in NYC and LA teenagers. Indoor home sources accounted for > 40% of the risk for compounds that were the major contributors of personal risk. These included formaldehyde, acetaldehyde, chloroform, and 1,4-dichlorobenzene. Contributions to personal risk from benzene, MTBE, tetrachloroethylene, carbon tetrachloride, and TCE were largely from outdoor sources. For most VOCs, at least a third of the personal risk came from other microenvironments. In LA there was a greater contribution from nonhome environments compared with NYC. The contributions to personal risk from indoor sources were minimal for the elements, with most elements having > 50% contribution from outdoor sources. A noted exception was chromium in NYC, which had a large contribution (53%) from other microenvironments. In LA, contributions from other microenvironments were also high for beryllium (57%) and nickel (66%).

### Comparison with U.S. EPA NATA-modeled risks

Estimated risks from the TEACH study were compared with estimated U.S. EPA risks that are based on ambient concentrations (U.S. EPA 2001). Comparing risks estimated from outdoor measured concentrations to risks from U.S. EPA–modeled concentrations can potentially increase confidence in the modeled results. Also, comparing measured personal risks to the U.S. EPA–modeled risk confirms the usefulness of using modeled concentrations to estimate personal risks. U.S. EPA–modeled concentrations were available from 1996 emissions inventories for most compounds, except for 1,4-dichlorobenzene, for which only the 1990 values were available (U.S. EPA 2001). Modeled concentrations for ethylbenzene and styrene were not available.

**Figure 4:**
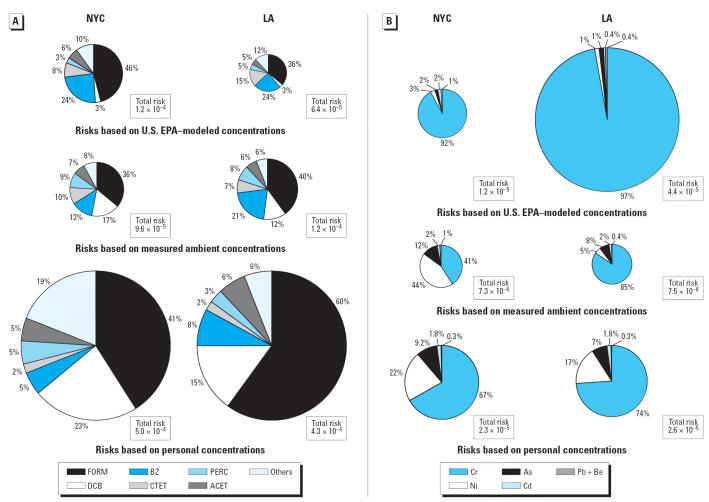
Distribution of cumulative excess cancer risks from VOCs (*A*; does not include contributions from ethylbenzene and styrene) and elements (*B*), based on median U.S. EPA–modeled, TEACH ambient (home outdoor), and TEACH personal concentrations in NYC and LA. The size of the pie chart is proportional to the cumulative risk. The cumulative risks are given in the boxes next to the pie charts, and represent excess cancer risk. For example, 1.2 × 10^−4^ is about 1 excess cancer case expected to develop per 10,000 people. Abbreviations for VOCs: ACET, acetaldehyde; BZ, benzene; CTET, carbon tetrachloride; DCB, 1,4-dichlorobenzene; FORM, formaldehyde; PERC, tetrachloroethylene; Others, carbon tetrachloride, methylene chloride, and TCE. Elements: As, arsenic; Be, beryllium; Cd, cadmium; Cr, chromium; Ni, nickel; Pb, lead.

The comparison of median risk values from the U.S. EPA model, TEACH outdoor home concentrations, and TEACH personal concentrations are shown as pie charts in Figure 4A and B, for VOCs and elements, respectively. The size of the pie chart is proportional to the total risk, and each segment shows compound specific contributions. We used U.S. EPA median cumulative risk estimates for NYC (including New York, Kings, Bronx, and Queens Counties) and for LA County (U.S. EPA 2001). In NYC, the U.S. EPA and TEACH outdoor risk estimates were similar (120 vs. 96 per million, respectively). For LA, measured TEACH ambient concentrations yielded risk estimates that were twice as high as U.S. EPA–modeled estimates (120 vs. 64 per million, respectively). Both the modeled and the measured median ambient concentrations greatly underestimated median personal VOC risks in both NYC and LA. The overall cumulative risks differed by more than a factor of 5 compared with U.S. EPA–modeled estimates in NYC and by almost a factor of 7 in LA.

**Figure 5:**
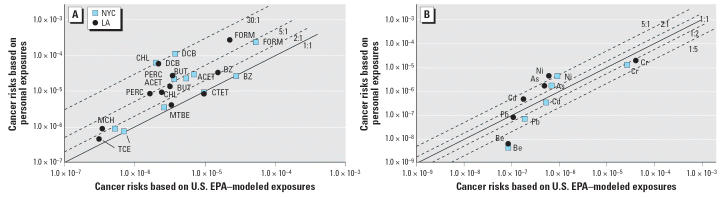
Comparison of U.S. EPA–modeled concentrations and TEACH personal upper-bound cancer risk for VOCs (*A*) and elements (*B*). Abbreviations for VOCs: ACET, acetaldehyde; BUT, 1,3-butadiene; BZ, benzene; CHL, chloroform; CTET, carbon tetrachloride; DCB, 1,4-dichlorobenzene; FORM, formaldehyde; MCH, methylene chloride; PERC, tetrachloroethylene. Abbreviations for elements: As, arsenic; Be, beryllium; Cd, cadmium; Cr, chromium; Ni, nickel; Pb, lead. Cancer risks are upper-bound lifetime estimates; for example, 1 × 10^−6^ is one excess cancer case in 1 million people.

Formaldehyde accounted for the greatest portion of the total modeled, ambient, and personal risk, contributing around 40%, climbing to 60% for personal risk in LA. Benzene was a significant portion of the risk for modeled and ambient exposures (~ 20%) but was < 10% for personal exposures. In contrast, 1,4-dichlorobenzene contributed < 5% in the U.S. EPA model but was significantly greater for personal exposures. The ratios between the U.S. EPA–modeled risks and the measured personal risks for each VOC are plotted in Figure 5A. Benzene, carbon tetrachloride, MTBE, and TCE risk estimates were comparable to U.S. EPA model estimates. As shown in the present study, these compounds have predominantly ambient sources; thus, the modeled estimates adequately predict risks from compounds with ambient sources. In contrast, compounds with predominantly indoor source contributions—chloroform and 1,4-dichlorobenzene—accounted for the largest difference in risks between personal and U.S. EPA–modeled concentrations.

The results for modeled, ambient, and personal concentrations for elements are compared in Figure 4B. Elemental data show that modeled median concentrations were higher than median measured ambient concentrations, and more so in LA (approximately six times higher) than in NYC (~ 60% higher). Estimated cumulative risks from personal exposures were a factor of 2 higher than modeled concentrations for the elements in NYC. The opposite was seen in LA, where the median risks from the U.S. EPA model were twice as high as the personal risk estimates.

The results for the elements show very different distributions of risk depending on the source of the data. Modeled concentrations show a dominant contribution of risk by chromium (> 90%) in both cities. The risks based on ambient concentrations show a different pattern in LA and NYC. In NYC, there was a high contribution of nickel (44%) followed by chromium (41%), whereas in LA chromium dominated (85%), even though the total risks were about equal. Based on the personal concentrations, chromium dominated with a contribution of about 70%, followed by about a 20% contribution from nickel in both cities. As shown in Figure 5B, U.S. EPA–modeled concentrations overestimated the risks from chromium and beryllium but underestimated the risks from nickel and arsenic in both cities.

## Discussion

We determined the carcinogenic risks associated with both personal exposures and measured outdoor concentrations of a suite of VOCs and particle-bound elements for a sample of high school students in NYC and LA. The results were compared with U.S. EPA risk estimates that were based on outdoor modeled concentrations. We also considered what compounds and factors contributed to the differences in risks between individual teenagers across the two cities.

For VOCs but not metals, the U.S. EPA risks based on modeled outdoor concentrations were a fairly good predictor of the TEACH risks based on measured outdoor home concentrations, particularly for the students living in NYC. Teenagers in NYC were recruited from a magnet school that drew students from several boroughs of the city, and therefore outdoor home measurements were taken from a fairly representative sample across the New York metropolitan area.

In contrast, the U.S. EPA–modeled concentrations underpredicted measured concentrations and risks in LA. The TEACH LA teenagers were recruited from a local high school in the south central area, and all students lived within 5 km of the school. Unlike for the NYC sample, ambient home measurements were not located across the much larger LA County.

For specific VOCs, only 1,4-dichlorobenzene had significantly higher measured than modeled concentrations. This difference may be due to sources not accounted for in the U.S. EPA source model, changes in emissions between the modeled and monitored years, or an artifact of our sampling. Most participants lived in apartment buildings or multifamily buildings, and outdoor samples were collected using a meter-long boom extending from the window. Thus, outdoor samples may have been influenced by indoor sources. For elements, greater differences were observed for LA than in NYC, potentially because homes were less representative of the region.

The cancer risk estimates from personal concentrations exceed values based on outdoor measured and U.S. EPA–modeled concentrations for VOCs in both cities. This large difference was due to the significant contribution from compounds with predominantly indoor sources, including formaldehyde, 1,4-dichlorobenzene (especially in the highest exposed individuals), and acetaldehyde, and in NYC, chloroform. Interestingly, only chloroform differed significantly between cities. Indoor sources of chloroform include use of chlorinated water. Concentration differences may be due to differences in drinking water treatment processes (e.g., chlorination, filtration to reduce organic matter, and ozonation) or differences in water use (City of Los Angeles 2004; Principe et al. 2000). For compounds of predominantly outdoor origin, benzene contributed significantly to the personal risks. Outdoor sources of benzene are mainly from vehicle traffic, and reductions of benzene in gasoline have contributed to a substantial decrease in benzene levels. Indeed, Wallace (1991) found mean personal concentrations of benzene to be 15 μg/m^3^, 3-fold higher than the personal levels found in this study.

The median cumulative carcinogenic risks associated with personal exposures to VOCs were 666 per million population in NYC and 486 per million in LA, assuming a lifetime exposure. The cumulative values for the elements were an order of magnitude lower, 23 and 26 per million in NYC and LA, respectively. Direct comparisons with other studies of HAPs are difficult because different studies include different compounds and risk estimates are not always based on personal exposures. For example, a risk assessment by Pratt et al. (2000) yielded a maximum cancer risk of 47 per million population based on monitored ambient concentrations in Minnesota, lower than our findings in part because of regional differences, but also because fewer compounds were included. In contrast, an estimate by Woodruff et al. (2000) across all of the United States yielded a median cancer risk estimate of 180 per million population, whereas median risks in California were found to be 270 per million (Morello-Frosch et al. 2000). These studies were both based on modeled ambient concentrations but included compounds not measured in the TEACH study, such as polycyclic aromatic hydrocarbons (PAHs).

A few studies have determined cancer risks from measured personal exposures. A recent cancer risk assessment was conducted by Payne-Sturges et al. (2004) of a cohort in Baltimore, Maryland. The authors estimated a much lower median cumulative risk of 120 per million but did not include formaldehyde and 1,4-dichlorobenzene, two compounds that contributed significantly in our estimate. Tancrede et al. (1987) used personal VOC Total Exposure Assessment Methodology (TEAM) data for New Jersey to evaluate cancer risks and found a much elevated risk of 19,000 per million, mostly because the authors included all compounds in their cancer assessment, assigning a cancer potency value to compounds with no published values.

Another evaluation of the TEAM data from six urban areas was conducted by Wallace (1991), who considered risks only from compounds with published cancer potency values. The resulting risk of 837 per million was similar to our estimates (Wallace 1991). When comparing contributions from compounds, TEACH had a higher contribution for 1,4-dichlorobenzene and lower contributions from benzene and formaldehyde (not measured directly in TEAM but estimated from typical literature values at the time). Indoor levels of formaldehyde have likely decreased due to reductions in use of urea-formaldehyde foam insulation, a major indoor source. Indoor levels remain high, however, because of contributions from indoor sources such as cabinetry, doors, plywood subflooring, particleboard, numerous consumer products, and reactions of ozone with various surfaces (Brown 1999; Reiss et al. 1995; Weschler et al. 1992).

On the other hand, the carcinogenesis of formaldehyde remains controversial because of the unusual metabolism in rodents (Wallace 1991). McCann et al. (1986) compared cancer potencies derived using different statistical methods and found a large difference in risk estimates for formaldehyde (3.7 × 10^−6^ to 9,500 × 10^−6^), depending on the method used. He attributed the differences to the nonlinear cancer dose–response curve found for rats. Conolly et al. (2003) recently determined a much reduced cancer potency of formaldehyde using a biologically motivated model that assumes a threshold.

In addition to comparing personal cancer risk estimates to U.S. EPA–modeled estimates and estimates from other studies, we were also interested in determining which factors contributed to differences in risks across the teenage populations in the two cities, specifically, whether high-risk individuals were at higher risk because they were exposed to higher concentrations of multiple compounds or to a different group of compounds, than were lower-risk individuals. To address this, we assessed the risks from individual compounds in low-risk versus high-risk individuals.

For VOCs, 1,4-dichlorobenzene contributed more significantly in the higher risk groups. Indoor 1,4-dichlorobenzene sources primarily include mothballs and room deodorizers, and the use of these products likely contributes to increased risks. Adgate et al. (2004) found higher indoor levels of 1,4-dichlorobenzene among Hispanic and Pacific Islander study participants compared with other races and ethnicities. We explored potential differences in personal cumulative risks based on race in NYC, because approximately half the teenagers were African American and half were from Hispanic origin (data not shown), but did not find a significant difference in the distributions. However, a visual inspection of the cumulative distribution for 1,4-dichlorobenzene did suggest higher risks for the Hispanic teenagers in the upper 20th percentile of the population distribution. Beginning in 2006, California is banning the sale of solid deodorizers that contain 1,4-dichlorobenzene in recognition of possible risks from exposure to this compound (California Air Resources Board 2004).

The risks from particle-bound elements were significantly lower than the risks from VOC exposures and were driven largely by chromium in both cities. Source contributions for chromium were predominantly from outdoor and other microenvironments. In NYC, chromium contributed 75% of the total risk for the teenagers in the highest risk categories. A possible explanation may be higher exposures to chromium from commuting on the subway, as has been shown by Chillrud et al. (2004). In LA, the risk contributions from chromium were equally distributed across quartiles of participants, indicating possible exposures to similar sources. It is also noteworthy that the U.S. EPA–modeled concentrations for chromium tend to overestimate the risks associated with this element.

An exploratory survey of differences in exposures to particle-bound elements between Hispanic and African-American teenagers in NYC showed greater risk contributions in the Hispanic population for lead, cadmium, and nickel (data not shown). For chromium, however, we found higher risks in the African-American population. This is again substantiated by the findings of Chillrud et al. (2004), who showed a correlation between high chromium exposures and commuting by subway. Indeed, we found that of our sample population of African-American teenagers in NYC, 70% commuted by subway with an average commute time of 120 min, whereas only 45% of teenagers of Hispanic origin commuted by subway with an average commute time of 75 min.

This risk analysis is not a comprehensive analysis of all potential carcinogenic compounds. Of particular importance to cancer risks are PAHs. Morello-Frosch et al. (2000) found that exposures to outdoor sources of organic matter accounted for 34% of the cumulative cancer risk. Because we did not consider PAHs and many other carcinogens in our analysis, the cancer risks may be underestimated.

Conversely, several inherent limitations to this risk analysis could overestimate the cancer risks. Of the top five compounds with the highest cancer risk, only benzene is classified by the U.S. EPA as a known human carcinogen (U.S. EPA 2005b). There are also many known uncertainties associated with the inhalation unit risks. The toxicity data derived from animal studies have uncertainty associated with extrapolations from high doses used in animals to low human exposures. Also, extrapolating from animals to humans provides additional uncertainty. Data collected from occupational studies have uncertainty associated with high occupational exposures and also because occupational cohorts may not be representative of the overall human population. Unfortunately, the U.S. EPA does not provide confidence intervals for its cancer potency estimates, and thus, it is difficult to determine the relative magnitude of these uncertainties. In general, cancer potencies are upper-bound estimates that assume a lifetime (70 years) of exposures; however, our data represent only a “snapshot” of this exposure that varies over a lifetime. The new guidelines for cancer risk assessment were published by U.S. EPA in March 2005, and as cancer potency values are revised according to these new guidelines, we may begin to be able to elucidate the extent of these uncertainties (U.S. EPA 2005a).

Given the level of uncertainty associated with cancer risk assessments, it is perhaps useful to compare the cumulative risks estimated in this study to risks from other environmental exposures such as radon and passive smoking. We found that the VOC risk estimates from the TEACH analysis were in the same order of magnitude as risks from radon and passive smoking. Exposures to radon can result in cancer risks of about 1,000 per million, and the cancer risk associated with passive smoking has been estimated to be about 2,000 per million (Wallace 1991). However, because these risk estimates are based on epidemiological studies, they are considered somewhat more certain.

It is also of interest to place these risks in the context of the actual risks of getting cancer. The total lifetime cancer risk is approximately 1 in 3, and the risk of actually getting lung cancer (probably the most relevant cancer) is approximately 1 in 1,000, which corresponds to a lifetime risk (70 years) of about 1 in 14 (U.S. Cancer Statistics Working Group 2005). Therefore, the cumulative risk from exposure to VOCs in this study (~ 1 in 1,000) accounts for about 1.4% of the total lung cancer risks in the United States.

## Conclusions

Cancer risk estimates based on personal exposures can be used as a guide to help prioritize research and, as indicators of potential hazards, guide regulatory actions. For this population of urban-dwelling teenagers, exposure to indoor and other microenvironmental sources of 1,4-dichlorobenzene and formaldehyde contributed significantly to overall excess cancer risks from exposures to VOCs, and these risks were generally underestimated by U.S. EPA model predictions. Particle-bound elements, on the other hand, were associated with risks that were several orders of magnitude lower than for the VOCs, and exposures were mostly from outdoor sources and in some cases other microenvironments.

As the field of risk assessment evolves and more biologically plausible mechanisms are incorporated into risk assessments, we may find that the picture changes dramatically. There is also a need for more comprehensive risk analyses that incorporate a greater number of compounds, particularly PAHs, that have been shown to be important contributors to risk in other studies.

## Correction

In Table 2, for VOCs, values in the rows for MTBE and cumulative risk are incorrect in the original manuscript published online; they have been corrected here. In Table 3, some of the values under NHAPS have been changed.

## Figures and Tables

**Table 1:** Descriptive statistics of personal, indoor, and outdoor home concentrations averaged across season.

		Personal					Indoor home				Outdoor home			
Compound	No.^a^	% > LOD	Med	Mean ± SD	Min	Max	% > LOD	Med	Mean	Max	% > LOD	Med	Mean	Max
NYC														
VOCs (μg/m^3^)														
1,3-Butadiene	41 (25)	61	0.75	0.97 ± 1.15	ND	5.25	65	0.50	1.01	9.02	18	ND	0.13	1.99
1,4-Dichlorobenzene	41 (25)	100	9.68	41.7 ± 72.5	2.05	313	95	8.23	75.0	1,452	77	2.24	4.59	34.1
Acetaldehyde	46 (31)	100	12.8	17.4 ± 14.0	1.23	86.0	100	13.1	16.7	91.8	100	3.24	3.41	7.50
Benzene	41 (25)	93	3.27	3.82 ± 2.38	1.28	12.3	83	2.75	3.64	20.7	49	1.45	1.82	4.93
Carbon tetrachloride	41 (25)	100	0.58	0.60 ± 0.10	0.47	0.92	98	0.59	0.60	0.89	97	0.59	0.58	0.86
Chloroform	41 (25)	100	2.67	2.85 ± 1.90	0.39	8.43	100	2.40	2.96	8.17	62	0.17	0.24	1.99
Ethylbenzene	41 (25)	100	1.92	2.48 ± 1.91	0.85	9.79	100	1.65	2.48	17.9	100	1.08	1.33	5.29
Formaldehyde	46 (31)	100	17.1	21.4 ± 12.6	1.50	55.1	100	15.6	17.7	46.0	98	3.08	3.53	7.35
Methylene chloride	41 (25)	90	1.88	6.90 ± 23.3	0.31	150	83	1.77	9.02	176	44	0.82	1.35	12.9
MTBE	41 (25)	100	12.9	20.0 ± 33.6	4.81	224	100	12.0	18.8	185	97	10.3	12.5	73.0
Styrene	41 (25)	100	0.83	1.22 ± 1.12	0.43	6.16	93	0.79	0.98	2.94	21	0.29	0.30	0.65
Tetrachloroethylene	41 (25)	100	3.69	8.75 ± 17.6	1.31	104	100	3.24	6.60	78.3	97	1.46	4.69	87.1
Trichloroethylene	41 (25)	90	0.36	1.78 ± 5.60	ND	32.8	83	0.33	0.90	19.4	67	0.20	0.24	0.73
Elements (ng/m^3^)														
Arsenic	40 (0)	100	0.40	0.45 ± 0.37	0.18	2.57	100	0.35	0.40	1.06	100	0.29	0.37	0.78
Beryllium	40 (0)	98	0.0017	0.002 ± 0.0008	0.00027	0.0036	97	0.0014	0.0015	0.0034	96	0.0025	0.0028	0.011
Cadmium	45 (30)	100	0.18	0.25 ± 0.21	0.07	1.09	100	0.15	0.17	0.77	98	0.12	0.14	0.40
Chromium	39 (0)	90	1.04	1.99 ± 1.98	0.14	7.82	67	0.50	0.55	1.35	38	0.35	0.44	2.15
Lead	45 (30)	100	5.34	46.5 ± 248	2.14	1,667	100	5.02	12.6	198	100	5.31	6.49	19.8
Nickel	45 (30)	62	16.3	28.7 ± 52.8	1.62	353	48	15.7	23.7	348	30	19.2	21.3	94.3
LA														
VOCs (μg/m^3^)														
1,3-Butadiene	40 (31)	80	0.30	0.47 ± 0.46	ND	1.89	68	0.34	0.41	1.47	25	ND	0.12	1.70
1,4-Dichlorobenzene	40 (31)	93	5.27	36.6 ± 72.8	1.03	341	88	6.19	47.4	261	60	1.80	2.65	12.2
Acetaldehyde	41 (34)	100	11.4	14.6 ± 7.94	6.57	39.4	100	11.7	13.0	35.9	100	3.76	3.83	6.03
Benzene	40 (31)	100	4.16	4.64 ± 1.80	2.68	11.27	100	3.30	3.87	11.4	100	3.14	3.32	5.56
Carbon tetrachloride	40 (31)	100	0.55	0.56 ± 0.10	0.26	0.76	100	0.52	0.52	0.75	100	0.54	0.53	0.68
Chloroform	40 (31)	95	0.36	0.47 ± 0.42	0.04	2.55	93	0.45	0.63	4.19	20	0.07	0.07	0.19
Ethylbenzene	40 (31)	100	3.13	3.96 ± 2.28	1.76	11.7	100	2.35	2.75	8.55	100	2.46	2.45	4.91
Formaldehyde	41 (34)	100	20.5	22.4 ± 8.62	12.0	52.2	100	17.5	19.3	58.9	100	3.97	4.08	7.62
Methylene chloride	40 (31)	100	1.84	2.40 ± 1.66	0.50	8.85	100	1.64	2.00	6.01	98	0.90	1.06	3.96
MTBE	40 (31)	100	15.3	17.4 ± 7.23	9.80	40.6	100	13.3	15.5	44.7	100	14.8	15.7	30.4
Styrene	40 (31)	100	1.13	1.26 ± 0.48	0.52	2.80	98	0.98	1.10	2.38	85	0.60	0.65	1.27
Tetrachloroethylene	40 (31)	100	2.24	2.64 ± 1.31	1.07	6.13	100	1.79	2.04	5.66	100	1.54	1.69	3.12
Trichloroethylene	40 (31)	83	0.22	0.26 ± 0.16	0.08	0.81	66	0.18	0.22	0.83	45	0.11	0.12	0.45
Elements (ng/m^3^)														
Arsenic	41 (32)	100	0.40	0.42 ± 0.13	0.17	0.71	100	0.42	0.44	0.81	100	0.42	0.43	0.79
Beryllium	41 (32)	100	0.0027	0.003 ± 0.003	0.00089	0.017	98	0.0015	0.0018	0.0045	98	0.0013	0.0018	0.0060
Cadmium	41 (32)	100	0.26	0.28 ± 0.10	0.08	0.59	100	0.24	0.29	1.72	100	0.24	0.32	1.70
Chromium	41 (32)	98	1.59	2.16 ± 2.18	0.39	13.47	98	1.25	1.41	3.08	100	1.52	3.02	58.2
Lead	41 (32)	100	6.48	8.37 ± 9.66	2.80	64.4	100	6.99	14.1	223	100	6.94	11.3	132
Nickel	41 (32)	100	18.2	23.4 ± 22.0	0.99	124	100	4.17	6.56	42.5	100	4.78	6.71	29.7

Abbreviations: Max, maximum; Med, median; Min, minimum; MTBE, methyl-*tert* butyl ether; ND, not detected.

a Total number of participants’ samples (number of participants with a paired sample in the second season).

**Table 2:** Upper-bound excess cancer risks for NYC and LA TEACH participants based on personal exposures.

	WOE				NYC				LA			
Compound	IRIS	IARC	Unit risk	Source	Mean	Median	90th percentile	Max	Mean	Median	90th percentile	Max
VOCs												
1,4-Dichlorobenzene	NC	2B	1.1 × 10^−5^	CalEPA	458	106	1,049	3,440	403	58.0	1,065	3,754
Formaldehyde	B1	2A	1.3 × 10^−5^	IRIS	278	223	529	716	291	267	391	679
Chloroform	B2	2B	2.3 × 10^−5^	IRIS	65.6	61.4	112	194	10.9	8.2	22.3	58.6
Tetrachloroethylene	NC	2A	5.9 × 10^−6^	CalEPA	51.6	21.8	89.8	615	15.6	13.2	28.6	36.2
Acetaldehyde	B2	2B	2.2 × 10^−6^	IRIS	38.4	28.2	76.7	189	32.2	25.1	50.4	86.7
Benzene	A	1	7.8 × 10^−6^	IRIS	29.8	25.5	45.0	96.0	36.2	32.4	55.9	87.9
1,3-Butadiene	B2	2A	3.0 × 10^−5^	IRIS	29.0	22.4	66.8	158	14.0	9.1	34.7	56.6
Carbon tetrachloride	B2	2B	1.5 × 10^−5^	IRIS	9.06	8.70	11.2	13.9	8.4	8.2	10.2	11.4
TCE	B2,C	2A	2.0 × 10^−6^	CalEPA	3.56	0.72	2.81	65.5	0.52	0.43	0.97	1.62
MTBE	NC	3	2.6 × 10^−7^	CalEPA	5.20	3.37	6.41	58.3	4.52	3.97	7.18	10.6
Methylene chloride	B2	2B	4.7 × 10^−7^	IRIS	3.24	0.88	3.80	70.5	1.13	0.86	2.27	4.16
Ethylbenzene	NC	2B	5.0 × 10^−7^	CEP	1.24	0.96	1.88	4.90	1.98	1.56	3.19	5.87
Styrene	B2,C	2B	5.0 × 10^−7^	CEP	0.61	0.42	1.39	3.08	0.63	0.57	1.00	1.40
Cumulative risk					957	666	1,539	4,156	806	486	1,449	4,344
Elements												
Chromium VI	A	1	1.2 × 10^−2^	IRIS	23.8	12.5	57.9	93.8	26.0	19.1	36.8	162
Nickel, refinery dust	A	1	2.4 × 10^−4^	IRIS	6.88	3.90	10.7	84.7	5.61	4.38	11.2	29.8
Arsenic, inorganic	A	1	4.3 × 10^−3^	IRIS	1.93	1.71	2.62	11.0	1.79	1.72	2.51	3.07
Lead, inorganic	B2	2A	1.2 × 10^−5^	CalEPA	0.56	0.064	0.18	20.0	0.10	0.08	0.11	0.77
Cadmium	B1	1	1.8 × 10^−3^	IRIS	0.45	0.33	0.82	1.95	0.51	0.47	0.75	1.06
Beryllium	B1	1	2.4 × 10^−3^	IRIS	0.0043	0.0041	0.0068	0.0086	0.0081	0.0064	0.014	0.041
Cumulative risk					34.2	23.3	76.0	135	34.0	26.1	48.4	173

Abbreviations: IARC, International Agency for Research on Cancer; IRIS, Integrated Risk Information System; Max, maximum; WOE, weight of evidence. Data from CalEPA 2002; IRIS (U.S. EPA 2005b); CEP (Caldwell et al. 1998). Cancer risks are presented as excess cancers per million population.

**Table 3:** Hours per day spent in different microenvironments for teenagers in NYC and LA.

Microenvironment	NYC winter (*n* = 38) (mean ± SD)	NYC summer (*n* = 41) (mean ± SD)	LA winter (*n* = 41) (mean ± SD)	LA fall (*n* = 34) (mean ± SD)	NHAPS (mean)
Home	17.1 ± 2.3	19.7 ± 4.1	16.8 ± 2.4	19.2 ± 3.9	16.5
School	6.3 ± 2.8	0.1 ± 0.5	7.4 ± 2.3	3.7 ± 3.4	NA
In other	1.1 ± 1.3	3.9 ± 3.2	0.9 ± 1.6	1.4 ± 1.8	2.6
Out other	1.0 ± 0.8	1.4 ± 1.3	1.0 ± 0.8	1.5 ± 1.8	1.8
Subway	0.9 ± 0.8	0.6 ± 0.7	0.0	0.0	NA
Car/bus	0.5 ± 0.5	0.5 ± 0.5	0.5 ± 0.5	0.9 ± 0.8	1.3

Abbreviations: NA, not available; NHAPS, National Human Activity Patterns Survey (national overall mean *n* = 9,153; Kleipeis et al. 2001).

## References

[b1-ehp0114-001558] Adgate JL, Church TR, Ryan AD, Ramachandran G, Fredrickson AL, Stock TH (2004). Outdoor, indoor, and personal exposure to VOCs in children. Environ Health Perspect.

[b2-ehp0114-001558] Akland GG (1993). Exposure of the general-population to gasoline. Environ Health Perspect.

[b3-ehp0114-001558] Brown SK (1999). Chamber assessment of formaldehyde and VOC emissions from wood-based panels. Indoor Air.

[b4-ehp0114-001558] Calderon J, Ortiz-Perez D, Yanez L, Diaz-Barriga F (2003). Human exposure to metals. Pathways of exposure, biomarkers of effect, and host factors. Ecotoxicol Environ Safety.

[b5-ehp0114-001558] Caldwell JC, Woodruff TJ, Morello-Frosch R, Axelrad DA (1998). Application of health information to hazardous air pollutants modeled in EPA’s Cumulative Exposure Project. Toxicol Ind Health.

[b6-ehp0114-001558] CalEPA 2002. Air Toxics Hot Spots Program Risk Assessment Guidelines Part II: Technical Support Document for Describing Available Cancer Potency Factors. Berkeley, CA:California Environmental Protection Agency, Office of Environmental Health Hazard Assessment, Air Toxicology and Epidemiology Section.

[b7-ehp0114-001558] California Air Resources Board 2004. Final Statement of Reasons for Rulemaking, Including Summary of Comments and Agency Responses. Public Hearing to Consider Adoption of Proposed Amendments to the California Consumer Products Regulations and Method 310 and Adoption of Airborne Toxic Control Measure for para-Dichlorobenzene. Sacramento:California Air Resources Board. Available: http://www.arb.ca.gov/regact/conprod/fsor.pdf [accessed 10 May 2006].

[b8-ehp0114-001558] Caussy D, Gochfeld M, Gurzau E, Neagu C, Ruedel H (2003). Lessons from case studies of metals: investigating exposure, bioavailability, and risk. Ecotoxicol Environ Safety.

[b9-ehp0114-001558] Chillrud S, Epstein D, Ross J, Sax S, Pederson D, Spengler J (2004). Elevated airborne exposures of teenagers to manganese, chromium, and iron from steel dust and New York City’s subway system. Environ Sci Technol.

[b10-ehp0114-001558] City of Los Angeles 2004. Water Quality: City of Los Angeles Water Services Annual Water Quality Report. Available: http://www.ladwp.com/ladwp/cms/ladwp001965.jsp [accessed 10 May 2006].

[b11-ehp0114-001558] Clayton CA, Pellizzari ED, Whitmore RW, Perritt RL, Quackenboss JJ (1999). National human exposure assessment survey (NHEXAS): distributions and associations of lead, arsenic and volatile organic compounds in EPA Region 5. J Expos Anal Environ Epidemiol.

[b12-ehp0114-001558] Clean Air Act Amendments of 1990 1990. Public Law 101-549.

[b13-ehp0114-001558] Conolly RB, Kimbell JS, Janszen D, Schlosser PM, Kalisak D, Preston J (2003). Biologically motivated computational modeling of formaldehyde carcinogenicity in the F344 rat. Toxicol Sci.

[b14-ehp0114-001558] Kinney PL, Chillrud SN, Ramstrom S, Spengler JD (2002). Exposures to multiple air toxics in New York City. Environ Health Perspect.

[b15-ehp0114-001558] Klepeis NE, Nelson WC, Ott WR, Robinson JP, Tsang AM, Switzer P (2001). The National Human Activity Pattern Survey (NHAPS): a resource for assessing exposure to environmental pollutants. J Expo Anal Environ Epidemiol.

[b16-ehp0114-001558] McCann J Horn L Gitman J Nero AV 1986. Potential Risks from Exposure to Organic Carcinogens in Indoor Air. Report LBL-22473. Berkeley, CA:Lawrence Berkeley Laboratory.

[b17-ehp0114-001558] Morello-Frosch RA, Woodruff TJ, Axelrad DA, Caldwell JC (2000). Air toxics and health risks in California: the public health implications of outdoor concentrations. Risk Anal.

[b18-ehp0114-001558] Payne-Sturges DC, Burke TA, Breysse P, Diener-West M, Buckley TJ (2004). Personal exposure meets risk assessment: a comparison of measured and modeled exposures and risks in an urban community. Environ Health Perspect.

[b19-ehp0114-001558] Pratt GC, Palmer K, Wu CY, Oliaei F, Hollerbach C, Fenske MJ (2000). An assessment of air toxics in Minnesota. Environ Health Perspect.

[b20-ehp0114-001558] Principe MA Stasiuk WN Stern IA 2000. Protecting New York City’s drinking water sources. In: Proceedings for American Planning Association: National Planning Conference, April 2000, New York City, NY. Available: http://www.asu.edu/caed/proceedings00/PRINCIP/princip.htm [accessed 25 July 2006].

[b21-ehp0114-001558] Reiss R, Ryan PB, Koutrakis P, Tibbetts SJ (1995). Ozone reactive chemistry on interior latex paint. Environ Sci Technol.

[b22-ehp0114-001558] Sax S 2003. Evaluation of Exposures and Cancer Risks from Volatile Organic Compounds among Inner-City Teenagers [PhD thesis]. Boston:Harvard School of Public Health, Department of Environmental Health.

[b23-ehp0114-001558] Sax SN, Bennett DH, Chillrud SN, Kinney PL, Spengler JD (2004). Differences in source emission rates of volatile organic compounds in inner-city residences of New York City and Los Angeles. J Expos Anal Environ Epidemiol.

[b24-ehp0114-001558] Tancrede M, Wilson R, Zeise L, Crouch EAC (1987). The carcinogenic risk of some organic vapors indoors: a theoretical survey. Atmos Environ.

[b25-ehp0114-001558] U.S. Cancer Statistics Working Group 2005. United States Cancer Statistics: 2002 Incidence and Mortality. Atlanta, GA:Centers for Disease Control and Prevention and National Cancer Institute.

[b26-ehp0114-001558] U.S. EPA 1999a. Determination of Volatile Organic Compounds in Ambient Air using Active Sampling onto Sorbent Tubes. Cincinnati, OH:Office of Research and Development, U.S. Environmental Protection Agency.

[b27-ehp0114-001558] U.S. EPA 1999b. Determination of Formaldehyde in Ambient Air using Adsorbent Cartridge Followed by High Performance Liquid Chromatography (HPLC): Active Sampling Methodology. Cincinnati, OH:Office of Research and Development, U.S. Environmental Protection Agency.

[b28-ehp0114-001558] U.S. EPA 2001. National-Scale Air Toxics Assessment for 1996. Research Triangle Park, NC:Office of Air Quality Planning and Standards, U.S. Environmental Protection Agency. Available: http://www.epa.gov/ttn/atw/nata [accessed 10 May 2006].

[b29-ehp0114-001558] U.S. EPA 2005a. Guidelines for Carcinogen Risk Assessment. Washington, DC:U.S. Environmental Protection Agency.

[b30-ehp0114-001558] U.S. EPA 2005b. Integrated Risk Information System (IRIS). Washington, DC:U.S. Environmental Protection Agency. Available: http://www.epa.gov/iris/index.html [accessed 10 May 2006].

[b31-ehp0114-001558] Wallace L 1987. The Total Exposure Assessment Methodology (TEAM) Study. Washington, DC:Office of Research and Development, U.S. Environmental Protection Agency.

[b32-ehp0114-001558] Wallace L (1991). Comparison of risks from outdoor and indoor exposure to toxic chemicals. Environ Health Perspect.

[b33-ehp0114-001558] Wallace LA, Pellizzari ED, Hartwell TD, Davis V, Michael LC, Whitmore RW (1989). The influence of personal activities on exposure to volatile organic compounds. Environ Res.

[b34-ehp0114-001558] Wallace LA, Pellizzari ED, Hartwell TD, Whitmore RW, Zelon H, Perritt RL (1988). The California TEAM study: breath concentrations and personal exposures to 26 volatile compounds in air and drinking water of 188 residents of Los Angeles, Antioch, and Pittsburg, CA. Atmos Environ.

[b35-ehp0114-001558] Weisel CP (2002). Assessing exposure to air toxics relative to asthma. Environ Health Perspect.

[b36-ehp0114-001558] Weschler CJ, Hodgson AT, Wooley JD (1992). Indoor chemistry: ozone, volatile organic compounds, and carpets. Environ Sci Technol.

[b37-ehp0114-001558] Woodruff TJ, Axelrad DA, Caldwell J, Morello-Frosch R, Rosenbaum A (1998). Public health implications of 1990 air toxics concentrations across the United States. Environ Health Perspect.

[b38-ehp0114-001558] Woodruff TJ, Caldwell J, Cogliano VJ, Axelrad DA (2000). Estimating cancer risk from outdoor concentrations of hazardous air pollutants in 1990. Environ Res.

[b39-ehp0114-001558] Woolfenden EA McClenny WA 1997. Compendium of Methods for the Determination of Toxic Organic Compounds in Ambient Air: Method TO-17. Cincinnati, OH:U.S. Environmental Protection Agency.

